# A statistical analysis of murine incisional and excisional acute wound models

**DOI:** 10.1111/wrr.12148

**Published:** 2014-03-17

**Authors:** David M Ansell, Laura Campbell, Helen A Thomason, Andrew Brass, Matthew J Hardman

**Affiliations:** 1 The Healing Foundation Centre, Faculty of Life Sciences, University of ManchesterManchester, United Kingdom; 2 Department of Computer Sciences, University of ManchesterManchester, United Kingdom

## Abstract

Mice represent the most commonly used species for preclinical in vivo research. While incisional and excisional acute murine wound models are both frequently employed, there is little agreement on which model is optimum. Moreover, current lack of standardization of wounding procedure, analysis time point(s), method of assessment, and the use of individual wounds vs. individual animals as replicates makes it difficult to compare across studies. Here we have profiled secondary intention healing of incisional and excisional wounds within the same animal, assessing multiple parameters to determine the optimal methodology for future studies. We report that histology provides the least variable assessment of healing. Furthermore, histology alone (not planimetry) is able to detect accelerated healing in a castrated mouse model. Perhaps most importantly, we find virtually no correlation between wounds within the same animal, suggesting that use of wound (not animal) biological replicates is perfectly acceptable. Overall, these findings should guide and refine future studies, increasing the likelihood of detecting novel phenotypes while reducing the numbers of animals required for experimentation.

Cutaneous wounds heal via sequential overlapping processes, which have been extensively documented by Werner & Grose and Shaw & Martin^1^,^2^ and others. Mice remain the most widely used species for preclinical in vivo wound healing studies by some margin (Supporting Information Table S1). Although alternative species, such as pig, are reported to more closely mirror human healing,^3^ mice with their smaller size, ease of use, and availability of transgenic strains, are almost certain to continue as the model of choice for mechanistic wound healing research.^4^ Surprisingly, to the best of our knowledge, there is no consensus over the optimum acute wound model, dimensions, or method of evaluation for mouse studies (Supporting Information Table S1).

Excisional wounds are most common in the literature, but numerous methodological variations exist: (1) the size of excision (and number of wounds per animal)—from 2 mm diameter up to as much as 20 mm diameter; (2) the tools employed to generate the wound—biopsy punches lacerate, surgical scissors crush, lasers cauterize^5^; and (3) occlusive dressings or splints^6^ or nonocclusive bandages of varying composition will lead to changes in the normal wound environment influencing healing.^7^ Incisional wounds are the second most frequently employed model, with generally more consistency across publications: wounds range from 10 to 15 mm in length, are generally full thickness and scalpel induced. However, around one-third of studies employ suture to close the wound margins. Intriguingly, the size, periodicity, and type of suture employed has been shown to significantly alter tensile forces across a sheet of wounded skin.^8^–^10^

To complicate matters further, a variety of different parameters are used to quantify wound progression, such as size, strength, reepithelialization, inflammatory response, a range of molecular and biochemical markers, and level of scarring. When monitoring overall healing outcome, noninvasive macroscopic wound planimetry (using serial daily photographs of the same animal) is regularly used to plot a temporal wound reduction profile. Unfortunately, it remains unclear how faithfully this measurement reflects changes in individual repair processes, such as inflammation or matrix deposition that cannot be visualized externally. Histological analysis is the “gold standard” method to obtain this information; however, this requires the animal be culled precluding serial measurements.

It is clearly beyond the scope of any one group to compare experimentally the myriad of potential wounding strategies. Instead, in this study, we perform a detailed, highly powered, within biological replicate comparative evaluation of the two most common secondary intention wound types: incisional and excisional. Healing outcome was assessed in each mouse via both planimetric and histological methods. Moreover, reanalysis of preexisting archived mouse wound tissue allowed comparison of intramouse and intergroup variability. This experimental approach allowed us to ask crucial, currently unanswered, questions relating to optimal study design:

(1) Which experimental model (incisional vs. excisional) is least variable?
(2) Which analysis method (planimetry vs. histology) is least variable?
(3) How similar are two wounds from the same individual (versus across individuals)?
(4) What is an optimal experimental group size for murine wounding studies?

Finally, armed with the answers to these questions, we outline optimal experimental design with respect to: (1) wound model; (2) analysis methodology; (3) group size; and (4) statistical analysis of data (individual wounds vs. individual mice).

## Methods

### Which model (incisional vs. excisional) is least variable?

**Figure 1:**
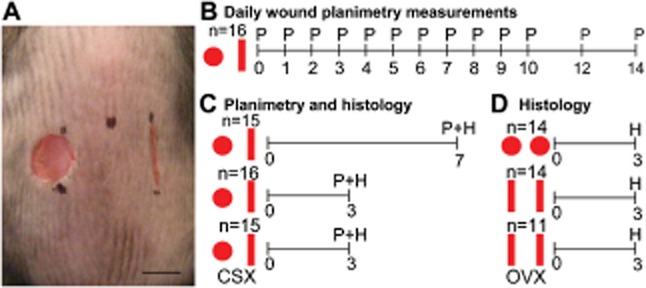
Experimental design allowing evaluation of incisional and excisional wounds within an individual. (A) Macroscopic image (immediately after wounding) indicates experimental design. (B–D) Schematic time lines depict measurements taken from each cohort of animals on each day, *n* = number of mice per group. (B) Daily planimetric analysis, (C) planimetric and histological analysis at a single time point or, (D) histological analysis from archived tissue. (P) planimetry, (H) histology, (CSX) castration, (OVX) ovariectomy. Bar = 5 mm.

To determine the relative merits of incisional vs. excisional wounds, one wound of each type was generated within the same animal. Seven- to eight-week-old male C57/Bl6 mice were anesthetized by isofluorane inhalation, and the dorsal flank was shaved and sterilized with an alcohol swab (Alcotip, Shermond, Leicester, United Kingdom). One full-thickness circular 6 mm biopsy punch excision (surface area of 28.27 mm^2^) and one linear 1 cm scalpel incision were made 1 cm apart on the dorsum of mice in the telogen stage of hair cycle and left to heal by secondary intention (Figure 1A). Postoperatively, mice were housed individually to minimize wound disruption with access to food and water ad libitum. All experiments were reviewed and approved by the University of Manchester animal use committee and were conducted in accordance with United Kingdom home office regulations.

Macroscopic wound photographs were captured at the time of wounding and on each subsequent day (Finepix S5700 camera, Fujifilm, Bedford, United Kingdom) and area quantified (image pro plus software) to determine temporal healing profile (Figure 1B; *n* = 16). Additional mouse groups were collected at days 3 and 7 with wounds excised, bisected (laterally) using sharp scissors, and processed for histological analysis (Figure S1A). Hematoxylin & eosin staining was performed on tissue sections taken at the epicenter of the wound. Images were captured (Eclipse E400 microscope and spot insight camera, Nikon, Kingston Upon Thames, United Kingdom) and analyzed using image pro plus software. Wounds width was determined by measuring the distance between wound margins. Wound area was calculated from wound margins below the eschar, extending to the level of the panniculus carnosus. Reepithelialization was expressed as a percentage of full closure (Figure S1B). Figure 1B–C outlines experimental design. Signal-to-noise ratio (mean divided by standard deviation) was used as a measure of data robustness with bootstrapping performed to provide an estimate of variability. For cross-methodological analysis, histological wound width, area, and % reepithelialization data were combined with corresponding single time point planimetric measurement in mouse groups collected at days 3 and 7 (Figure 1C). Male mice surgically castrated at 6 weeks and wounded 2 weeks later, provided day 3 planimetry and histology data.

### Which method (planimetry vs. histology) is least variable?

In addition to histology/planimetry correlation data (see above), the % difference in the group signal-to-noise ratios (histology to planimetry) was determined for width and area measurements across both wound models at days 3 and 7.

### How similar are two wounds from the same individual (compared with across individuals)?

Within animal variability was assessed by linear correlation of histological area for an incisional and an excisional wound on the same animal (Figure 1C). In addition archived control female day 3 wound tissue from our previous work,^11^,^12^ two equivalent wounds per mouse, was reanalyzed (Figure 1D).

To assess independence, we randomly allocated a wound (i.e., left or right) from each mouse into two groups (A and B). The standard deviations (σ) of group A and B were independently calculated (i.e., each wound as a biological replicate) and a mean σ determined (indicating linkage). The random value was determined using the calculation: 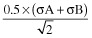


Our observed value was calculated as the σ of the average of both wounds from each individual (i.e., each mouse as a biological replicate). Comparison of our observed values with the expected linked or random values above indicates the degree of linkage. Further to this, we assessed the normal distribution of each data set (wound vs. mouse replicate). Assuming a normal distribution, central limit theorem predicts a convergence toward the mean of 1 standard deviation when using biological replicates, if the two wounds are indeed behaving completely independently.^13^

### What is an optimal group size?

Mean and standard deviation values derived from histological wound areas of female intact and an additional cohort of day 3 incisional wounded ovariectomized mice (Figure 1D) were used in a series of sample size power calculations for hypothetical experimental wound studies. The alpha and beta errors were set at 5% and 20%, respectively.

## Results

### Question 1: which model (incisional vs. excisional) is least variable?

#### For wound planimetry excisional wounds are less variable

**Figure 2:**
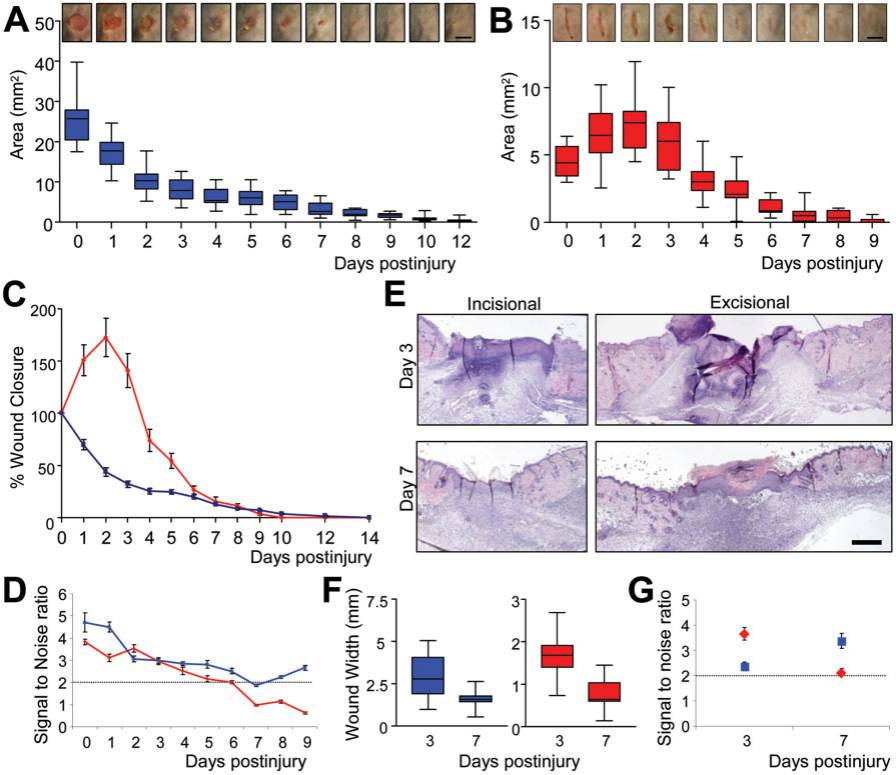
Planimetric wound closure is more reproducible in excisional wounds. Planimetric time course reveals differing temporal healing profiles for excisional and incisional wounds. Box and whisker plots map the changes in total wound size over time for excisional (A) and incisional (B) wound closure. Between wound divergence of healing profile is emphasized when data are expressed as percentage wound closure (C); with signal to noise ratio (D) indicating excisional wounds are more stable over time. (E) Histological analysis of excisional and incisional wounds at two distinct time points (day 3 and 7) illustrates the reduction in wound width over time for both wounds (F) mirroring planimetry. However, by histology, incisional wounds have a greater signal to noise ratio at day 3, while this is reversed at day 7 (G). Bootstrapping was performed to estimate S/N variability. Blue lines/squares, excisional data; Red lines/diamonds, incisional data. Data shown as mean ± SEM (C) or ± SD (D, G) (*n* = 15–16). Bar = 5 mm (A and B) and 500 μm (E).

The macroscopic planimetric time course is commonly used to evaluate excisional wound healing,^14^,^15^ yet is never applied to incisional wounds. As the scientific basis for this preference is unclear, we generated wound time course data for our incisional/excisional cowounded mice (*n* = 16). Intriguingly, incisional and excisional wounds show very different healing profiles when assessed planimetrically. Incisional wounds increase in area over the first 2 days of healing indicating variable expansion of the wound margins (Figure 2A and B). By contrast, excisional wounds decrease in area over the same period with less variability. Plotting the data as percentage wound area reduction accentuates the differences between models and illustrates the high variability at early stages of incisional repair (Figure 2C). These early differences are reflected in the bootstrapped wound signal-to-noise ratio, which is high and stable over an excisional wound time course but falls rapidly in incisional measurements (Figure 2D). This is true whether data are untransformed (not shown), normalized to the maximal wound area (not shown) or normalized to the initial wound size (Figure 2D).

Two further groups of mice were sacrificed at either day 3 or 7 postwounding to histologically assess difference between incisional and excisional models. Intriguingly, the clear planimetric advantage of excisional wounds fails to translate into histological assessment (Figure 2E–G), where wound width reduction is similar in both models (Figure 2F).

#### Correlation between planimetric and histological measurements is greater in incisional wounds

It is assumed that planimetry and histology measurements will correlate; however, our signal-to-noise ratio data across these two types of measurements (Figure 2G) appear at odds with this assumption. To our knowledge, this question has not been addressed in detail. Thus, we quantified within wound correlation of planimetry and histology from the same wound. As histological assessment of healing can be stratified into constituent processes (i.e., width, area and reepithelialization measurements), we compared each of these individually with planimetric data. While the terminal nature of histological analysis required planimetry of new mouse groups, these data mapped closely to the presented time course (Figure 2), indicating continuity between experiments.

**Figure 3:**
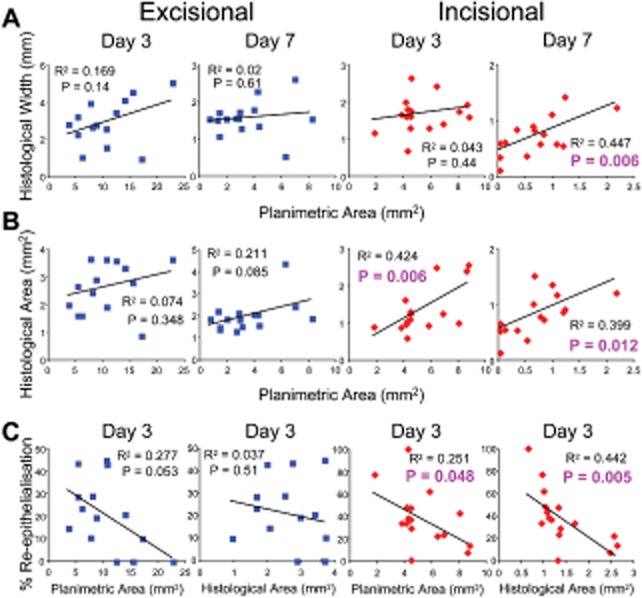
Histology and planimetry measurements fail to correlate for excisional wounds. Within wound linear regression reveals correlation between planimetric and histological measurements for incisional wounds at days 3 and 7. By contrast, no correlation is observed within excisional wounds. (A) Linear regression between planimetric area and histological width reveals a significant correlation in incisional wounds at day 7 (*p* = 0.006). (B) Correlation between planimetric area and histological area is significant only in incisional wounds at both days 3 (*p* = 0.006) and 7 (*p* = 0.012). (C) Histology-derived % reepithelialization at day 3 inversely correlated with planimetric area or histological area only in incisional wounds.

We observed significant correlation between macroscopic (planimetry) and microscopic (histology) measurements of the same incisional wounds at both day 3 (area: *R* = 0.42 and *p* < 0.01) and day 7 (width: *R* = 0.45 and *p* < 0.01; area: *R* = 0.40 and *p* < 0.02; Figure 3A and B). By contrast, excisional wounds showed little correlation between macroscopic and any microscopic measurements at either time point (Figure 3A and B). Parallel Spearman Rank correlation analysis (not shown) mirrored the presented linear correlation data. Planimetry has been proposed as a good surrogate measure of wound reepithelialization^16^ yet we observed statistically significant inverse correlation between reepithelialization and planimetry only in incisional wounds (Figure 3C *p* < 0.05) (Note: correlation between reepithelialization and planimetry was close to significance in the excisional model, *p* = 0.053). Collectively, widespread absence of correlation in the excisional model supports the use of incisional wounds for healing studies.

Finding: Excisional wounds are suited to planimetric analysis yet incisional wounds show far greater correlation between planimetric and histological parameters.

### Question 2: which method (planimetry vs. histology) is least variable?

#### Variability is substantially lower in histological measurements

**Figure 4:**
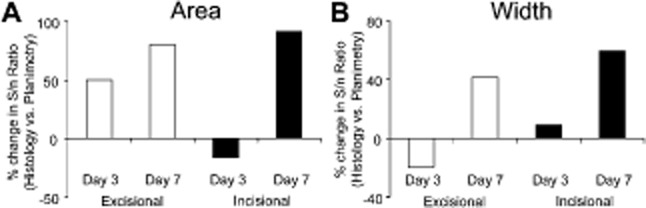
Variability is substantially lower in histological measurements. Histological measurements have a higher signal-to-noise (S/N) ratio across two different analyses when compared with planimetry. The percentage change in histological S/N ratio vs. planimetric S/N reveals comparatively greater histological S/N for both area (A) and width (B) measurements in 5 out of 8 comparisons. Little difference was observed in the remaining three groups. White bars, excisional wounds; black bars, incisional wounds.

In light of the above findings (i.e., the merits of the wound model depend on the method of analysis), we next asked which method (planimetry vs. histology) was preferable. To do this, we calculated signal-to-noise ratios for each wound type/time point (data presented as % change of signal-to-noise ratio comparing histology to planimetry; Figure 4). In addition, we generated a new planimetric measurement of wound width and carried out the corresponding comparison, i.e., “histology and planimetry” for two wound types (incision and excision) and at two time points (3 and 7 days) across two measurements (area and width). Collectively, these analyses reveal a substantially increased signal-to-noise ratio (less variable) for histological vs. planimetric measurements in five out of eight comparisons (with virtual equivalence in the remaining three; Figure 4).

Finding: As expected, histology outperforms planimetry at key wound time points.

### Combining questions 1 and 2: Which model/method combination is most predictive?

#### Incisional histology alone detects accelerated healing at day 3 in castrated mice

**Figure 5:**
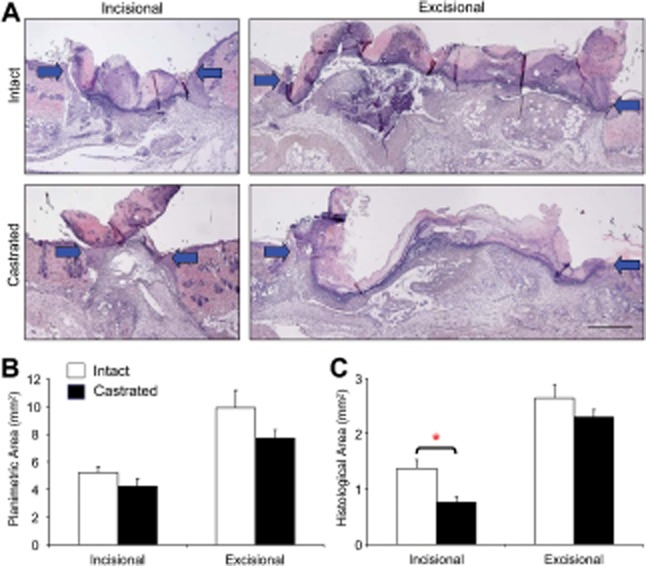
Incisional wound histology detects accelerated healing at day 3. Accelerated healing in castrated (CSX) mice is only detected in incisional wounds through histological analysis. (A) Representative histology depicts healing of incisional and excisional wounds in intact and CSX animals, arrows denote wound margins. (B) Planimetric analysis fails to show accelerated healing in CSX mice in either incisional or excisional wounds. (C) By contrast, histological wound area measurement shows accelerated healing (reduced histological area) only in incisionally wounded CSX mice. Data shown as mean ± SEM (*n* = 15–16). Bar = 500 μm. *p < 0.01.

In experimental studies, it is crucial to be able to reliably detect altered healing (vs. a control group). Therefore, we wounded castrated C57/Bl6 mice that have previously been shown to display accelerated repair.^17^ Using the wound design outlined in Figure 1A (i.e., both wound types in the same animal), we assessed day 3 incisional and excisional wound area both planimetrically and histologically in castrated vs. control mice. We find that histological analysis of incisional wounds is the only method able to show statistically altered healing in castrated mice (Figure 5C). All other combinatorial analysis showed a nonstatistically significant trend toward accelerated healing (i.e., no difference).

Finding: For this specific perturbation model and group size (*n* = 15) histological analysis of incisional wounds alone was able to demonstrate altered repair. Thus, in answer to questions 1 and 2, we suggest that incisional wounding and histological analysis should be the protocol adopted for future wounding studies.

### Question 3: How similar are two wounds from the same individual (versus across individuals)?

#### Wounds within the same mouse show high variability

**Figure 6:**
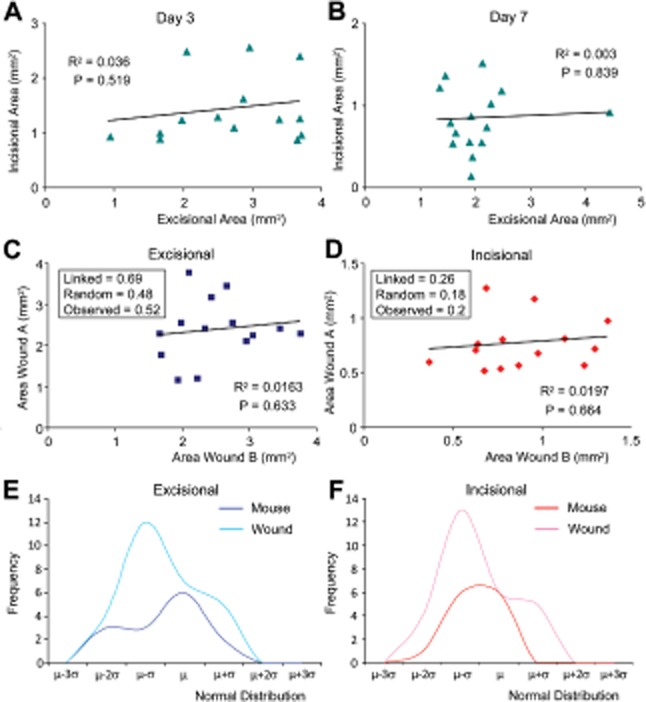
Wounds within the same animal show little correlation. (A–D) Scatter plots comparing histological areas of incisional and excisional wounds within individual mice, with associated *R*^2^ and *p*-values. No significant correlation was observed between incisional and excisional wounds on the same mouse at either day 3 (A) or day 7 (B). Further histological analysis of mice wounded with either two excisions (C) or two incisions (D) confirmed minimal relationship between wounds on the same animal (3 days postwounding). Box inserts (C and D) show the observed standard deviation is much closer to the standard deviation generated if the data are random rather than linked for either excisional or incisional wounds. (E and F) Normal distribution plots of wound biological replicates (*n* = 28, light color) vs. mouse biological replicates (*n* = 14, dark color) reveal a convergence toward the mean of 1 standard deviation when using each mouse as a biological replicate. This suggests for both excisions (E) and incisions (F) that the wounds behave independently to each other.

Discrepancy within the literature over whether mice or wounds should be used as biological replicates is extensive. To address which should be adopted as best practice, we determined the correlation between incisional and excisional wound size measured histologically within the same mouse. Intriguingly, we found no correlation at either day 3 or day 7, suggesting little if any mouse-specific influence (Figure 6A and B). The incisional and excisional models show very different overall healing profiles. To further address inter- and intragroup variability, we switched to our extensive tissue archive of female mice wounded with two wounds of the same type. Again, in both incisional and excisional models, we found no significant correlation between two wounds within a single experimental animal (Figure 6C and D). These data suggest that the two wounds are in fact independent. This was confirmed by the correlation coefficient (see methods), which indicated that two wounds within the same animal were only marginally more related than independent variables (see box Figure 6C and D). Spearman rank correlation analysis gave virtually identical results to this linear correlation data (not shown). Next, we assessed the normal distribution of our individual wounds (*n* = 28) vs. the mouse replicates (*n* = 14). Both incisional and excisional wounds displayed a convergence toward the mean of almost 1 standard deviation when analyzed as mouse replicates (Figure 6E and F). Central limit theorem would indicate that these wounds effectively behave as independent variables.^13^

Finding: Individual wounds within the same animal should be considered as independent biological replicates.

### Question 4: What is an optimal group size?

#### Power calculations reveal fewer mice are required if each wound is taken as a biological replicate

Using archived tissue comparing incisional wounds in control and ovariectomized (OVX) female mice, a model known to exhibit delayed wound repair,^18^,^19^ we performed power calculations to predict the number of mice required to detect healing phenotypes with a variety of hypothetical magnitudes of change (see Supporting Information Table S2). These clearly indicate that for changes in repair of 15–25% wound replicates become advantageous, necessitating fewer animals to detect a significant difference (see Supporting Information Table S2).

Finding: Using individual wounds as replicates reduces the number of mice required per experiment.

## Discussion

Until suitable nonsentient models are developed, which is unlikely in the foreseeable future, animal experimentation remains essential to the wound repair field. However, to our knowledge, a standardized acute wound model has yet to be agreed. In this study, we present an objective comparison of the benefits and limitations of the two most commonly used wounding models. Our findings suggest that simple changes in experimental design and analysis can yield more reproducible results, and thus allow more efficient screening of potential wound healing phenotypes.

First, our data reveal that while planimetry of excisional wounds follows a defined temporal healing profile, this shows little, if any, correlation to histological assessment. This surprising lack of correlation most likely reflects inherent differences in the measurements. Planimetry gives a two dimensional view of the wound surface, whereas histology measures both depth and width. Moreover, the eschar may partially obscure the wound causing planimetric underestimation of healing and increased variability, in line with our data (lower signal-to-noise ratio than histology). This finding is important for determining appropriate primary and secondary healing endpoints for future studies. In addition, histology provides more granularity to analyses, probing for apoptosis, proliferation, inflammation, or a range of other wound processes can pinpoint the mechanisms of altered repair.^20^,^21^ Histo-morphometric analysis can be employed to reveal subtle regional or cell-specific changes.^22^,^23^ We thus suggest that studies employing planimetry alone provide insufficient assessment of healing.

We acknowledge that it was not possible in this study to profile histologically every time point for which we present planimetric data. Indeed, in “real life” experiments, this would also be preclusive. Days 3 and 7 postwounding were specifically chosen as commonly used time points within the literature.^24^ Similarly, we chose a single model, castration, to confirm the discriminative ability of incisional histology. It is essential that predictivity is validated in additional models and we actively encourage replication by other groups. Our results clearly indicate that, assuming an appropriate time point is chosen, using both models (incision and excision) in the same study is superfluous. We suggest that investigators should inform reviewer(s) who stipulate use of their preferred model, that this is both unnecessary and inconsistent with the aims of the 3Rs.

While our data suggest that incisional wounds are preferable, we acknowledge that in some situations there are methodological advantages to the use of excisional wounds. First, the tissue excised during the wounding procedure can itself be used for histological or biochemical analysis providing a critical experimental control in some studies. For example; (1) where skin is pretreated prior to wounding this excised skin can be used to monitor potential induced morphological skin changes such as epidermal hyperplasia, inflammation, or altered hair cycling which would influence healing^25^,^26^; and (2) with increasing use of inducible genetically modified mouse strains, it is important to confirm successful gene knockdown/activation at the time of injury.^27^ In addition, any dressings, sutures, or topical vehicle may alter normal healing of either wound type^28^–^30^ and investigators will need to have a clear understanding of how any intervention away from the standard secondary intention model will affect their desired outcomes.

The high intramouse variability indentified in this study was particularly surprising. That two wounds within a single animal appear no more linked than between animals is an important finding. We are aware that advising the use of individual wounds from the same animal as replicates is controversial. Indeed, in our own previously published studies, we routinely take each dual wounded animal as a biological replicate (i.e., the mean of both wounds). Yet our data now clearly support wound replicates as a valid approach to improve the statistical power of any given study. Obviously, this would not be relevant when treatment(s) and control are within an animal, i.e., a complex factorial design.

Our statistical power calculations (Supporting Information Table S2) based on a large experimental data set should provide a useful reference for acute wound study design. In a carefully designed study with a defined acute model, time point(s) and analysis strategy, it should be possible to reliably detect between group differences in healing of 20% or greater, using mouse groups of single figures. However, for more subtle phenotypes, the numbers of mice required will outweigh the potential therapeutic benefit, rendering the experiment ethically questionable.

In conclusion, the data presented in this study provide clear support for the use of (1) incisional wounds and (2) histological analysis to quantify murine healing. Establishing optimal models, methodologies and analysis strategies for in vivo healing studies will “refine” existing protocols in order to “reduce” the numbers of animals used in future experiments, two of the founding principles of the 3Rs of animal research.

## Supporting Information

**Figure S1.** Histological measurements were taken using defined criteria. (A) Wound tissue was excised surrounded by approximately 5 mm of normal tissue, and bisected into two halves. Histological wound analysis was performed through the centre of the wound (blue dashed line). (B) Haematoxylin and Eosin stained sections were used to measure wound width (black double ended arrow). Wound area (blue area) was determined as the area of granulation tissue beneath the wound, excluding the eschar, and extending to the paniculus carnosus at each flank (blue arrows). Newly formed epidermis was measured (green dotted line), as was the non-healed portion (red dotted line), with degree of re-epithelialisation expressed as a percentage. Bar = 400 μm. **Table S1.** Murine in vivo wounding models published in 2010. **Table S2.** Power calculations estimate animal numbers required for hypothetical experiments.
