# Supplementary material for: A statistical analysis of murine incisional and excisional acute wound models
Source: Wound Repair Regen. 2014 Mar 17;22(2):281–7. doi: 10.1111/wrr.12148 (PMC4309476; doi:10.1111/wrr.12148)
Supplement: Supplementary file 1 — Figure S1. Histological measurements were taken using defined criteria. (A) Wound tissue was excised surrounded by approximately 5 mm of normal tissue, and bisected into two halves. Histological wound analysis was performed through the centre of the wound (blue dashed line). (B) Haematoxylin and Eosin stained sections were used to measure wound width (black double ended arrow). Wound area (blue area) was determined as the area of granulation tissue beneath the wound, excluding the eschar, and extending to the paniculus carnosus at each flank (blue arrows). Newly formed epidermis was measured (green dotted line), as was the non-healed portion (red dotted line), with degree of re-epithelialisation expressed as a percentage. Bar = 400 μm. Table S1. Murine in vivo wounding models published in 2010. Table S2. Power calculations estimate animal numbers required for hypothetical experiments. [file wrr0022-0281-sd1.pdf]

Table S1. Murine *in vivo* wounding models published in 2010.

| **Wound model** | **Publications** |
|---|---|
| Full thickness Excision secondary intention | 55 |
| Full thickness Excision (sutured) | 1 |
| Full thickness Excision (dressed) | 14 |
| Full thickness Excision (splinted) | 7 |
| Other excisional models | 7 |
| **Total excisions** | **84** |
| Full thickness incision secondary intention | 11 |
| Full thickness incision (sutured) | 5 |
| Other incisional models | 1 |
| **Total incisions** | **17** |
| **Total other wound models*** | **27** |

* including burn, laser, chemical, tape stripping

Table S2. Power calculations estimate animal numbers required for hypothetical experiments.

| | % difference in healing | | | | | | |
|---|---|---|---|---|---|---|---|
| | **5** | **10** | **15** | **20** | **25** | **30** | **50** |
| **Mouse replicates** | 144 | 36 | 16 | 9 | 6 | 4 | 1 |
| **Wound replicates** | 121 | 31 | 14 | 8 | 5 | 4 | 1 |

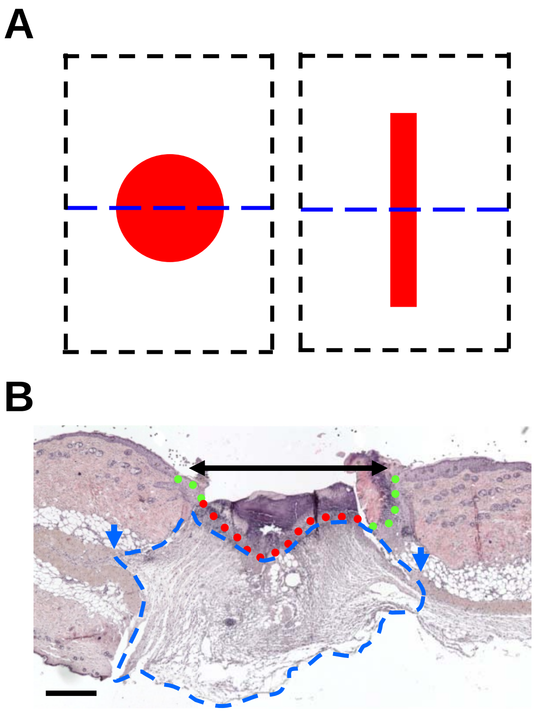


**Figure S1. Histological measurements were taken using defined criteria.** (A) Wound tissue was excised surrounded by approximately 5mm of normal tissue, and bisected into two halves. Histological wound analysis was performed through the centre of the wound (blue dashed line). (B) Haematoxylin and Eosin stained sections were used to measure wound width (black double ended arrow). Wound area (blue area) was determined as the area of granulation tissue beneath the wound, excluding the eschar, and extending to the paniculus carnosus at each flank (blue arrows). Newly formed epidermis was measured (green dotted line), as was the non-healed portion (red dotted line), with degree of re-epithelialisation expressed as a percentage. Bar = 400 μm.
